# Traffic Control of Bacteria-Derived Molecules: A New System of Host-Bacterial Crosstalk

**DOI:** 10.1155/2013/757148

**Published:** 2013-03-31

**Authors:** Hiroaki Konishi, Mikihiro Fujiya, Yutaka Kohgo

**Affiliations:** Division of Gastroenterology and Hematology/Oncology, Department of Medicine, Asahikawa Medical University, Asahikawa, Hokkaido 078-8510, Japan

## Abstract

Virulent microorganisms, such as pathogenic bacteria and viruses, are recognized by pattern recognition receptors (PRRs), including toll-like receptors (TLRs) and nucleotide-binding oligomerization-domain proteins (NODs), and induce inflammatory responses in mammalian hosts. Conversely, commensal bacteria and probiotics, which symbiotically confer health benefits on the host organisms, can lodge in the host intestinal tract without inducing intestinal inflammation. Recent advances in investigations concerning host-microbial interactions have shown that some effector molecules secreted from beneficial bacteria activate cell survival pathways, such as those mediated by p38 MAPK and Akt, and bring health benefits to mammalian hosts. It is noteworthy that such bacteria-derived molecules are taken into the intestinal epithelia through a transport or endocytosis system, thereafter exhibiting their beneficial effects. Understanding this traffic control process can aid in the comprehension of host and microbe interactions and may provide new insight to clarify the pathogenesis of intestinal disorders. This paper highlights the intestinal trafficking systems of bacteria-derived molecules that affect the bacterial functions and modulate epithelial signaling cascades. The latter mechanism may contribute to the maintenance of intestinal homeostasis by improving the host damage induced by virulence factors and various disease states.

## 1. Introduction

Pattern recognition receptors (PRRs), such as toll-like receptors (TLRs) and nucleotide-binding oligomerization-domain proteins (NODs), have been identified as sensors that recognize bacterial substances. Following the recognition of these substances, the receptors activate inflammation-related molecules, such as NF-*κ*B, and induce intestinal inflammation in order to protect intestinal tissue from being damaged by pathogenic bacteria [1]. However, more than 1,000 different commensal bacteria survive in the host intestines symbiotically, without inducing inflammatory responses. Some of these commensal bacteria and probiotics, which are considered to confer health benefits when administered in adequate amounts [2], also exhibit beneficial functions in the host intestines without inducing intestinal inflammation. This suggests that the host intestines recognize these beneficial bacteria through sensing systems which are distinct from those that recognize pathogenic bacteria. However, the systems that mediate the interactions between the host and beneficial bacteria have not yet been identified.

This paper highlights new insights concerning host-bacteria interactions that occur through the epithelial trafficking of bacteria-derived molecules, abnormalities of which are considered to be associated with the pathogenesis of intestinal disorders.

## 2. Intestinal Epithelia Sense Beneficial Bacteria through Different Systems from Those used for Pathogenic Bacteria

 The components and secretions of pathogenic bacteria, such as *Clostridium* and *Escherichia coli,* are recognized by host cells through the actions of TLRs and/or NODs, which activate inflammation-related molecules, such as NF-*κ*B, and induce inflammatory cytokines [3]. These sensing systems contribute to the recognition of abnormal intestinal conditions due to the invasion of pathogenic bacteria, thereby protecting intestinal tissues from attacks by these pathogens. 

Conversely, beneficial bacteria, including commensal bacteria and probiotics, symbiotically live in the host intestines and do not induce intestinal inflammation. Therefore, intestinal tissues are thought to possess other systems for sensing nonvirulent beneficial bacteria, although these systems have not yet been identified. Tao et al. proposed that live *Lactobacillus GG,* as well as its conditioned media, induces heat shock proteins in the mouse intestine and improves the barrier function of intestinal epithelia [4]. This suggests that some soluble factor(s) secreted by *Lactobacillus GG* mediate the beneficial functions of the probiotics. The conditioned media of other beneficial bacteria, including *Bacillus subtilis, Bifidobacterium breve, Lactobacillus plantarum,* and *Lactobacillus brevis,* also exert beneficial effects on the induction of cytoprotective proteins and the protection of the intestinal epithelia from oxidative stress and excess inflammation [5, 6]. These recent insights indicate that bacteria-derived molecules mediate interactions between the host and beneficial bacteria through novel sensing systems that may be different from those used for pathogenic bacteria. 

## 3. Intestinal Epithelia Possess Sensing Systems for Bacteria-Derived Molecules 

 Although it is known that beneficial bacteria function by secreting bacteria-derived molecules, these molecules have not been identified thus far. It is necessary to identify such bacteria-derived molecules in order to explore the sensing systems used for the beneficial bacteria in intestinal epithelia. This issue prompted researchers to elucidate and validate the effector molecules derived from beneficial bacteria, and four effector molecules have been identified from the conditioned media of bacteria. The conditioned media of *Bacillus subtilis* and *Lactobacillus brevis SBL88* were separated using several kinds of columns, and each fraction was tested for the ability to induce cytoprotective heat shock proteins, and consequently, competence and sporulation factor (CSF) and polyphosphate (poly P) were identified as effector molecules produced by *Bacillus subtilis* and *Lactobacillus brevis*, respectively [5, 6]. Independently, Yan et al. also identified two peptides produced by *Lactobacillus GG*, p75 and p40, as active components that possess antiapoptotic properties and activate cell survival via the Akt pathway [7]. These findings indicate that bacteria-derived molecules identified as soluble factors are secreted from bacteria and have roles as mediators of communication between the host intestines and bacteria. Furthermore, FITC-labeled CSF and immunostaining of poly P has been shown to be absorbed into human epithelial cell line Caco2/bbe cells over a short interval, suggesting that epithelial trafficking systems mediate the effects of beneficial bacteria [5]. The immunostaining demonstrated the capture of the p40 peptide by epithelial cells [8]. To clarify the mechanisms underlying such novel interactions between hosts and beneficial bacteria, it is necessary to determine each step of the sensing system by using bacteria-derived molecules. 

## 4. Bacteria-Derived Molecules Are Recognized by Epithelial Cells through Receptor-Mediated Systems

 In the mammalian intestine, many receptors are expressed, and their expression levels are strictly controlled for sensing intestinal bacteria. When pathogenic bacteria invade the digestive tract, these bacteria are recognized by PRRs through pathogen-associated molecular patterns (PAMPs), which are bacterial components (e.g., sugar chains, lipids, peptides, and nucleotides), and host cells then induce defense reactions, such as colitis and the activation of innate immunity, in response to these components. 

There are some probiotics which have been reported to exert their probiotic effects via PRR signaling. For example, probiotic *Escherichia coli* Nissle 1971 has anti-inflammatory effects that are mediated via the TLR2 and TLR4 pathways [9]. *Clostridium butyricum* is considered to be a probiotic bacterium that brings about its health benefits through NF-*κ*B activation via the TLR2 pathway [10]. The effects of *Lactobacillus plantarum* strain YU were partially mediated by TLR2 [11]. However, the ligands from beneficial bacteria which are recognized by PRRs have not been identified, and the mechanism of action after recognition via PRRs is still unclear. Even if the ligands secreted from beneficial bacteria are the same kinds of products produced by pathogenic bacteria, such as peptidoglycans and LPS, there might be some differences in these molecules that lead to differential signaling. When the ligands from beneficial bacteria bind to PRRs, the downstream activation of signaling might not be the same as when the receptors are activated by pathogenic bacteria. 

 Both p40 and p75 were identified as cytoprotective effector molecules from the cultured media of *Lactobacillus GG* [7]. *Lactobacillus casei* also has genetic information encoding p40 and p75, and these molecules induce the activation of the prosurvival EGFR-Akt pathway and have antiapoptotic effects [8, 12]. p40 fails to stimulate Akt activation when EGFR is inhibited or deleted in human colon cancer cell lines, an immortalized mouse colon epithelial (MCE) cell line or mouse colon tissue. FITC-labeled p40 treatment of mice leads to the accumulation of p40 in colon epithelial cells, especially in the proximal and middle parts of the colon, and immunostaining showed colocalization of p40 and phospho-EGFR. After the recognition of p40, the EGFR-Akt pathways are activated, which is a key step for promoting the proliferation of intestinal epithelial cells and for the antiapoptotic effects mediated through p40. These findings indicate that mammals recognize beneficial (as well as pathogenic) bacteria via receptors and that this helps to maintain the homeostasis of the intestinal environment. In the case of intestinal diseases, it has been strongly suggested that the tolerance of bacteria is broken by the expressional abnormalities and/or genetic mutation(s) of receptors. 

## 5. Bacteria-Derived Molecules Are Absorbed via Transporter-Mediated Trafficking Systems of Intestinal Epithelia

**Figure 1:**
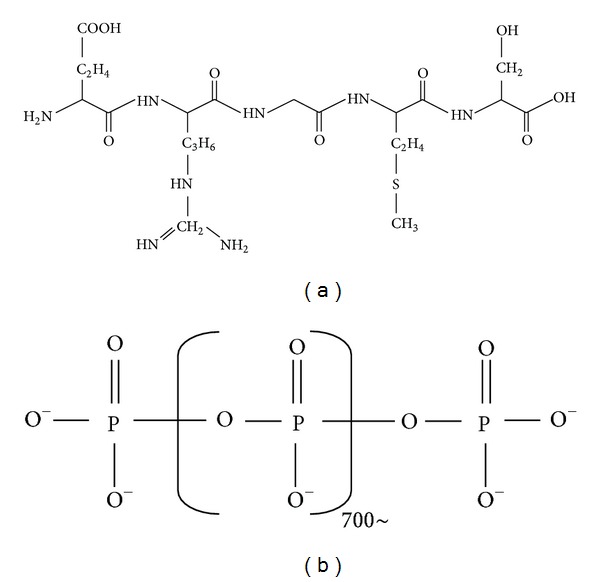
The structure of CSF and poly P. (a) CSF is pentapeptide, ERGMT, whose N-terminal is charged in positive. (b) poly P is long chain which consists of over 700 phosphates.

In the digestive tract, the transport system is thought to be strictly controlled to prevent invasion of antigens (such as food particles and bacteria) and to facilitate the uptake of import nutrients (such as amino acids and peptides) [13]. It is suggested that epithelial membrane transporters play important roles as the transport systems, which exist between the intestinal lumen and epithelia. Bacteria possess similar transport systems using transporters through the bacterial cell membrane that function to import nutrients as well as export small diffusible signal molecules, called quorum-sensing molecules, used for bacterial cell-to-cell communication [14]. CSF, which has been identified to be an effective molecule produced by *Bacillus subtilis,* as mentioned above, is a quorum-sensing molecule that controls the sporulation of bacteria [15]. Interestingly, CSF can be used for host and microbe communication, as well as bacterial communication, by its transport into the intestinal epithelia. Because CSF is a pentapeptide (ERGMT) that possesses a cationic charge in the N-terminal, as shown in Figure 1(a), organic cation transporters (OCTNs) are considered to be candidates for the transport of CSF, and the novel organic cation transporter 2 (OCTN2), a member of the OCTN family that is highly expressed in intestinal epithelia, was identified as the main transporter of CSF. The absorption of FITC and ^14^C-labeled CSF is dramatically decreased in OCTN2 siRNA-transfected caco2/bbe cells, indicating that OCTN2 plays a pivotal role in the epithelial trafficking of CSF. This suggests that mammals and bacteria can communicate with each other, beyond species, through the trafficking of bacterial products by mammalian epithelia.

However, it remains controversial whether these transporter trafficking systems are specific for communication between the host and beneficial bacteria. PEPT1 is an SLC transporter that accepts di/tripeptides and various peptide-mimics nonselectively but not free amino acids or peptides with more than three amino acid residues [32, 33]. The PEPT1 expression is high in the epithelial cells of the small intestine [34]. With respect to bacterial recognition, PEPT1 transports bacterial proinflammatory substances, such as small formylated bacterial peptide (fMLP), muramyl dipeptide (MDP), and Tri-DAP secreted from many enteric bacteria, including *Escherichia coli*, into intestinal epithelial cells [35]. These imports trigger NF-*κ*B signals and induce IL-8 and monocyte chemoattractant protein-1 (MCP-1). This activation is suspected to initiate intestinal inflammatory responses in inflammatory diseases [29]. The overexpression of PEPT1 enhances the sensitivity of the NOD2 signaling pathway that detects intracellular bacterial peptidoglycan-derived muramyl peptides [36]. In fact, this change in the PEPT1 expression pattern is reported to occur in patients with chronic ulcerative colitis (UC) and Crohn's disease (CD) [34] and in intestinal epithelial HT29-Cl.19A cells transfected by enteropathogenic *Escherichia coli* (EPEC) [37]. Accordingly, each transporter is thought to have a distinct affinity for the individual beneficial or pathogenic bacteria, which thereby modulate the inflammatory responses in the mammalian intestinal tract.

## 6. Bacteria-Derived Molecules Are Absorbed via Endocytic Trafficking Systems in Intestinal Epithelia

In mammals, endocytosis is an indispensable process by which cells takeup macromolecules, such as hydrophilic nutrients, soluble molecules, and bacteria, by forming vesicles derived from the plasma membrane. Endocytosis-related proteins, such as caveolin and clathrin, are recruited to ligands by binding to the molecules through cell surface proteins, such as integrins and receptors, and thus creating vesicles that coat the components [38]. Invaginated vesicles are recruited to endosomes, where the ligands are normally digested. Integrins accept various ligands into the extracellular matrix, and several integrins that experience endocytosis are recycled back into the plasma membrane and reused for endocytosis [39]. These are indispensable steps for antigen presentation, acquired immunity, and host homeostasis.

Bacteria-derived poly P, which we identified as an effective molecule produced by* Lactobacillus brevis*, forms a long chain containing over 700 phosphates (Figure 1(b)). Due to the large molecular weight of poly P, epithelial membrane transporters appear not to transport this molecule. It has been reported that poly P containing approximately 65 phosphates can bind to basic FGF2, thereby facilitating the binding of FGF-2 to its cell surface receptor and accelerating the growth of normal human fibroblast cells [40]. However, it is not clear how the long chain of poly P including over 700 phosphates is recognized by intestinal tissues.

**Figure 2:**
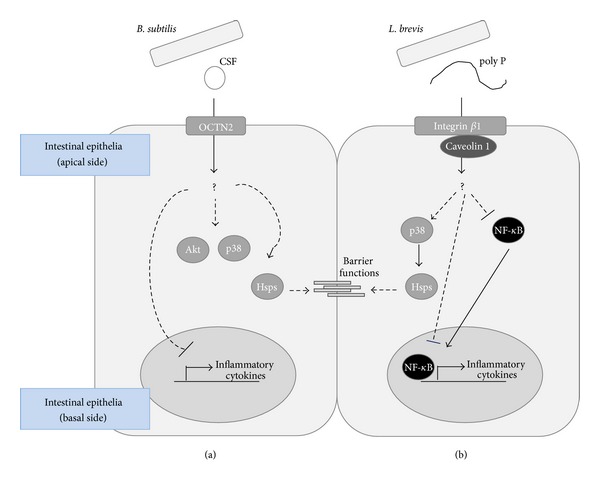
Bacteria-derived CSF and poly P are transported through different trafficking systems and exhibit cytoprotective and anti-inflammatory effects in human intestinal tissues. *Bacillus subtilis* secrets CSF, which is transported by epithelia cell membrane transporter OCTN2 (a). *Lactobacillus brevis* secrets poly P, which is captured by integrin *β*1 and absorbed by caveolin-dependent endocytosis (b). These trafficking systems mediate the augmentation of the intestinal barrier function and exhibit anti-inflammatory effects.

Long-chain poly P synthesized using biochemical methods with polyphosphate kinase has been assessed, and its interaction with integrin and the relationship with endocytosis have been clarified. The immunoprecipitation of the reaction mixture of ^32^P-labeled poly P and integrin *β*1 peptide, which binds with endocytosis-related caveolins, exhibited direct binding [6], and immunochemical methods showed that poly P is captured by integrin *β*1 in the intestinal plasma membrane and then is absorbed into the epithelial cells. The absorption of poly P was observed to dramatically decrease by integrin *β*1 inhibition, thus indicating that integrin *β*1-caveolin-dependent endocytosis is responsible for epithelial trafficking of poly P.  Figure 2(b) and the caveolin dependent recognition system in Table 1. This suggests that host and microbe communication is mediated by the trafficking of bacterial molecules through endocytosis as well as the transport of mammalian epithelia cells.

However, virulent bacteria as well as beneficial bacteria utilize endocytic pathways for invasion and infection. *Listeria monocytogenes* invades host cells using clathrin-dependent endocytosis and induces diseases such as meningitis [30]. Immunochemical methods have demonstrated that InlB, a surface molecule expressed by *Listeria monocytogenes,* interacts with hepatocyte growth factor, Met, and then monoubiquitinates Met by recruiting the ubiquitin ligase, Cbl. Clathrin is recruited to monoubiquitinated Met, and *Listeria monocytogenes* invades the mammalian cells. Enterotoxigenic *Escherichia coli* (ETEC) secretes a heat-stable, proteinaceous factor (not yet characterized) that blocks NF-*κ*B signaling by targeting I*κ*B*α* polyubiquitination. The conditioned media of ETEC prevent the activation of NF-*κ*B when intestinal epithelial cells are treated with tumor necrosis factor (TNF), interleukin-1*β*, and flagellin. This effect is diminished by the RNA interference-mediated knockdown of clathrin, but not by inhibition of caveolin, suggesting that this factor utilizes clathrin-dependent endocytosis to enter host cells [31]. Accordingly, endocytic pathways also appear to have a distinct affinity for individual production of beneficial or pathogenic bacteria, thereby modulating inflammatory responses in the mammalian intestinal tract.

## 7. Trafficking Systems Contribute to Intestinal Homeostasis and Improvement of Intestinal Disorders through the Intake of Bacteria-Derived Molecules

The trafficking systems of bacteria-derived molecules contribute to the activation of cell survival signals and the induction of cytoprotective proteins. CSF treatment activates p38 MAPK and Akt and induces heat shock protein 27 in caco2/bbe cells. Moreover, increases in the intestinal barrier function mediated by CSF have been shown in *in vitro* ^51^Cr releasing assays and *ex vivo* intestinal loop studies. The upregulation of the barrier function is diminished by OCTN2-specific siRNA, suggesting that the OCTN2-mediated trafficking system plays a pivotal role in conferring protection against oxidative stress.

The poly P treatment activates p38 MAPK, induces heat shock protein 27 in caco2/bbe cells, and increases the intestinal barrier function through its recognition via integrin *β*1, as assessed in *in vitro* immunostaining of E-cadherin and F-actin and *ex vivo* intestinal loop studies [6]. The increases in the barrier function are diminished by the inhibition of integrins or endocytosis by specific inhibitors or RNA interference. This indicates that trafficking of poly P is an indispensable step for activating cell survival signaling and p38 MAPK and protecting the intestinal epithelia from oxidative stress. However, it is not clear how poly P is released from vesicles before activating p38 MAPK and inducing heat shock protein 27. Furthermore, there is no evidence whether the effector molecules from bacteria are transported from epithelial cells to other cells. Clarifying the mechanisms underlying these effects and answering the many questions regarding the tolerance of bacteria may identify novel signaling pathways involved in host-bacterial crosstalk.

## 8. Therapeutic Effects of Bacteria-Derived Molecules on Intestinal Diseases

Inflammatory bowel diseases (IBD), including ulcerative colitis and Crohn's disease, are chronic and refractory inflammatory conditions that cause severe hemorrhage, intestinal perforation, and intestinal obstruction due to severe fibrotic changes. Although some therapeutic agents can relieve the activity of IBD, most IBD patients experience multiple relapses during their lives. Because the etiology of IBD has not been identified, surgeries such as intestinal resection and strictureplasty are frequently performed in patients with severe stenosis. In IBD patients, abnormal expression levels of heat shock proteins and populations of gut flora have been reported [41, 42]. Based on the basic research mentioned above, the cytoprotective and inflammatory effects of CSF and poly P, Figure 2, a preclinical trial using a dextran-sulfate-sodium (DSS-) and/or oxazolone-induced mouse colitis model, a representative model of IBD [43, 44], was used to assess the therapeutic effects of CSF, poly P, and p40. CSF and poly P improved colon length, the histological score of intestinal inflammation, the inflammatory cytokine expression, and the survival rate compared to those observed in the control mice [6, 45]. Moreover, poly P improved fibrotic changes in the mucosal and submucosal layers of the colon and the expressions of fibrosis-related molecules such as TGF-*β* and SMADs, illustrating that the use of poly P is feasible to relieve intestinal fibrotic changes in patients with IBD. p40 completely prevented the reduction in the colon length, intestinal inflammation, and apoptosis, which are induced by DSS- and oxazolone-induced colitis [8]. These findings indicate that bacteria-derived molecules are attractive targets for the development of new therapeutic agents.

## 9. Future Perspectives

To date, only a few reports have identified effector molecules derived from beneficial bacteria that have treatment effects for intestinal inflammation. Because large numbers of bacteria symbiotically live in the mammalian intestines, many unknown effectors are produced by these bacteria that may have activities for the treatment of intestinal diseases, even if those whose causes are unknown. As described in this paper, some groups have already conducted preclinical trials to cure intestinal inflammation using bacterial effector molecules (such as CSF, poly P, and p40). Not only CSF, poly P and p40 but also other as yet unknown bacterial molecules may be clinically applied as medicines in the near future. To clarify the mechanisms responsible for the effects of probiotic effector molecules derived from beneficial bacteria and the genetic factors that predict the efficacy of these molecules, genetically modified animals and genome-wide analyses of samples from individual patients will be needed. The polymorphisms of plasma membrane molecule genes such as *NOD2*, *OCTN2*, *MDR1,* and *integrin *β*7* have been reported to be susceptible to inflammatory bowel diseases [46–49]. The functions of CSF and poly P are mediated by OCTN2 transport and integrin *β* capturing, respectively. Therefore, made-to-order treatment can be established by analyzing the genetic backgrounds of patients with intestinal inflammation.

It has been reported that some probiotic bacteria such as *Bifidobacterium* and* Lactobacillus* possess antitumor effects [50, 51]. Such beneficial bacteria may release effector molecules that are transported into cancer cells through trafficking systems where they mediate the antitumor effects of bacteria. However, the responsible molecules and mechanisms underlying these antitumor effects have not been identified. If the molecules from beneficial bacteria that exhibit antitumor effects are identified, therapeutic agents that restore the body's natural state with little or no side effects may be developed.

As mentioned earlier in this paper, the findings of novel host-microbial interactions that function through the trafficking of bacteria-derived molecules in the mammalian intestinal epithelia indicate that transporter- and endocytosis-mediated pathways contribute to host and bacterial interactions through the actions of plasma membrane proteins, such as OCTN2 and integrins, by controlling the physiological functions of cells and monitoring intestinal homeostasis. These activities likely vary in different species and depend on particular strains of bacteria. New therapeutic agents with novel mechanisms related to such intestinal trafficking can be developed when bacteria-derived molecules possessing protective effects for the intestinal tissues are identified. 

## Figures and Tables

**Table 1:** Recognition systems for microorganisms.

Recognition systems				Functions	References
	Sensors	Effective molecules	Microorganisms
Receptors	TLR1/TLR2 TLR2/TLR6	Lipopeptide, lipoteichoic acid, peptidoglycan, lipoarabinomannan, lipoproteins	Gram-positive bacteria	Activation of NF-κB	[16–20]
	TLR3	Double-strand RNA	Virus	Induction of IFN	[21]
	TLR4	Lipopolysaccharide	Gram-negative bacteria	Activation of NF-κB	[20, 22]
	TLR5	Flagellin	Gram-negative bacteria which have flagellum	Activation of NF-κB	[23]
	TLR7	Single-stranded viral RNA	Virus	Activation of NF-κB	[24]
	TLR8	Single-stranded viral RNA	Virus	Activation of NF-κB	[25]
	TLR9	Bacterial CpG DNA	Bacteria	Activation of NF-κB	[26]
	NOD1	Mesodiaminopimelic acid	Gram-negative bacteria	Activation of NF-κB	[27]
	NOD2	Muramyl dipeptide	Gram-negative bacteria	Activation of NF-κB	[28]
	EGFR	p40, p75	*Lactobacillus GG *	Activation of Akt pathway	[8]
Transporters	OCTN2	CSF	*Bacillus subtilis *	Activation of Akt, p38 MAPK pathway	[5]
	PEPT1	fMLP, MDP, Tri-DAP	Gram-negative bacteria	Activation of NF-κB	[29]
Endocytosis	Clathrin	InlB	*Listeria monocytogenes *	Invasion to host mammal's cell	[30]
		?	Enterotoxigenic *Escherichia coli *	I*κ*B*α* Polyubiquitination	[31]
	Caveolin	poly P	*Lactobacillus brevis *	Activation of p38 MAPK pathway	Under submission
